# Molecular Characterization and Functional Analysis of the Nattectin-like Toxin from the Venomous Fish *Thalassophryne maculosa*

**DOI:** 10.3390/toxins14010002

**Published:** 2021-12-21

**Authors:** Monica Lopes-Ferreira, Ines Sosa-Rosales, Pedro Ismael Silva Junior, Katia Conceicao, Adolfo Luis Almeida Maleski, Leticia Balan-Lima, Geonildo Rodrigo Disner, Carla Lima

**Affiliations:** 1 Immunoregulation Unit of the Laboratory of Applied Toxinology (CeTICs/FAPESP), Butantan Institute, Vital Brasil Avenue, 1500 Butantan, Sao Paulo 05503-009, Brazil; adolfo.maleski@esib.butantan.gov.br (A.L.A.M.); leticia.lima@esib.butantan.gov.br (L.B.-L.); disner.rodrigo@gmail.com (G.R.D.); carla.lima@butantan.gov.br (C.L.); 2 Escuela de Ciências Aplicadas del Mar, Universidad de Oriente, Boca de Rio 6304, Venezuela; sosaines@gmail.com; 3 Protein Chemistry Unit of the Laboratory of Applied Toxinology (CeTICs/FAPESP), Butantan Institute, Vital Brasil Avenue, 1500 Butantan, Sao Paulo 05503-009, Brazil; pedro.junior@butantan.gov.br; 4 Peptide Biochemistry Laboratory, UNIFESP, Sao Jose dos Campos 12247-014, Brazil; katia.conceicao@unifesp.br; 5 Post-Graduation Program of Toxinology, Butantan Institute, Vital Brasil Avenue, 1500 Butantan, Sao Paulo 05503-009, Brazil

**Keywords:** *Thalassophryne*, nattectin, reverse-phase HPLC, MALDI-ToF, hemagglutinating activity, antibacterial activity, inflammation, toxinology, animal toxins

## Abstract

TmC4-47.2 is a toxin with myotoxic activity found in the venom of *Thalassophryne maculosa*, a venomous fish commonly found in Latin America whose envenomation produces an injury characterized by delayed neutrophil migration, production of major pro-inflammatory cytokines, and necrosis at the wound site, as well as a specific systemic immune response. However, there are few studies on the protein structure and functions associated with it. Here, the toxin was identified from the crude venom by chromatography and protein purification systems. TmC4-47.2 shows high homology with the Nattectin from *Thalassophryne nattereri* venom, with 6 cysteines and QPD domain for binding to galactose. We confirm its hemagglutinating and microbicide abilities independent of carbohydrate binding, supporting its classification as a nattectin-like lectin. After performing the characterization of TmC4-47.2, we verified its ability to induce an increase in the rolling and adherence of leukocytes in cremaster post-capillary venules dependent on the α5β1 integrin. Finally, we could observe the inflammatory activity of TmC4-47.2 through the production of IL-6 and eotaxin in the peritoneal cavity with sustained recruitment of eosinophils and neutrophils up to 24 h. Together, our study characterized a nattectin-like protein from *T. maculosa*, pointing to its role as a molecule involved in the carbohydrate-independent agglutination response and modulation of eosinophilic and neutrophilic inflammation.

## 1. Introduction

Lectins are a comprehensive group of proteins with carbohydrate-binding properties. Due to their binding singularity, many natural lectins, purified from several sources such as plants, algae, and fungi, have prospective applications in biotechnology, medical research, and crop protection. Additionally, many groups have identified lectins in organs and tissues (gills, eggs, electric organ, stomach, intestine, liver, skin, mucus, and plasma) of various fish species [1]. Lectins have been reported to have a wide array of functions, including immune-relevant ones such as pathogen recognition, agglutination, opsonization, complement activation, phagocytosis, and other functions such as splicing of RNA, protein folding, trafficking of molecules, control of cell proliferation, and roles in development [2,3].

Interestingly, our group has previously described a lectin, named Nattectin, in the venom gland of the fish *Thalassophryne nattereri* [4]. Nattectin is a basic monomeric protein, non-glycosylated, galactose-specific lectin from the C-type family, presenting remarkable pro-inflammatory activity. *T. nattereri* and *Thalassophryne maculosa* are venomous toadfish that belong to the family Batrachoididae. They are benthic ambush predators that favor from sandy or muddy substrates where their cryptic coloration, or the habit of burying under sand and mud, helps them avoid detection by their prey. Their omnivorous diet is composed of sea worms, crustaceans, mollusks, and other fish. They are found in temperate and tropical waters throughout the coast of America, Europe, Africa, and India. These species present one of the most adapted teleost venom apparatus composed of four canaliculated spines coupled to venom glands at their base capable of delivering a painful wound to predators. Thus, the venom apparatus has more of a defense function rather than predation.

Studies on *T. nattereri* envenomation and its venom composition have been carried out in Brazil by our group since 1998 at the Butantan Institute [5,6]. *T. nattereri* stands out among the venomous animals of medical importance in Brazil for the number of accidents it causes in the North and Northeast regions and the seriousness of the cases [7,8,9]. One of the main symptoms of *T. nattereri* envenomation is the immediate, intense pain that persists over 24 h. Erythema and edema are also shortly noticed with the efflorescence of bubbles with serous content. These lesions evolve to long-remaining necrosis with a delayed healing process devoid of specific drug treatment [10,11,12,13].

However, few studies have been conducted with *T. maculosa*, mainly found in Venezuela, Colombia, and the islands of Aruba, Curaçao, and Trinidad and Tobago. It is known that its venom presents a mixture of bioactive toxins differently expressed in females and males [14]. *T. maculosa* venom induces a significant necrotic lesion characterized by delayed neutrophil influx to the footpad of mice. An acute production of IL-1β, IL-6, and later secretion of TNF-α, MCP-1, KC, and mediators from arachidonic acid metabolism such as LTB4 and PGE2 were detected in the exudate of inflamed footpads [15]. In addition, we demonstrated that bone marrow-derived macrophages and dendritic cells were strongly stimulated by the venom, which demonstrates its ability to stimulate a specific and systemic immune response [16]. Furthermore, Sosa-Rosales et al. [17] performed a partial identification of two proteins with myotoxic activity in *T. maculosa* venom using reverse-phase HPLC and named them according to column retention time as TmC4-47.2. SDS-Page analysis of the crude venom showed a few weighty bands (one located above 97 Mw, one between 68 and 97 Mw, one major band between 29 and 43 Mw, and the last one located below 18.4 Mw). Then, it seems that the isolated nattectin-like protein is one of the main components of the venom, which includes a significant mixture of bioactive molecules involved in the local inflammatory lesion [17].

We understand that the advance in the knowledge of the pattern of ischemic and necrotic lesions induced by *T. maculosa* and the description of the main toxins can lead to the identification of targets for therapeutic intervention. Therefore, the study of the TmC4-47.2 toxin becomes an important tool for understanding the mechanisms of action involved in envenomation. In the present study, we performed the characterization of the TmC4-47.2 protein sequence using sequential C18 and C8 affinity column chromatography and mass spectrometry and determined its functions as a lectin.

## 2. Results

### 2.1. Purification of TmC4-47.2 Toxin from Thalassophryne maculosa Venom

Initially, the solution of *T. maculosa* venom chromatographed on a C18 column coupled to a high-pressure liquid chromatography system (Figure S1) generated 3 fractions, with the presence of the toxin mainly in fraction 2 that was subsequently re chromatographed 3 times on a reverse-phase C8 column. The first passage of fraction 2 through the C8 column generated 5 new fractions, with the toxin present in fractions 3, 4, and 5 (Figure S2). The second passage through the C8 column of the 3 previous pooled fractions generated 6 more fractions with the protein present only in fractions 3 to 5 (Figure S3).

**Figure 1:**
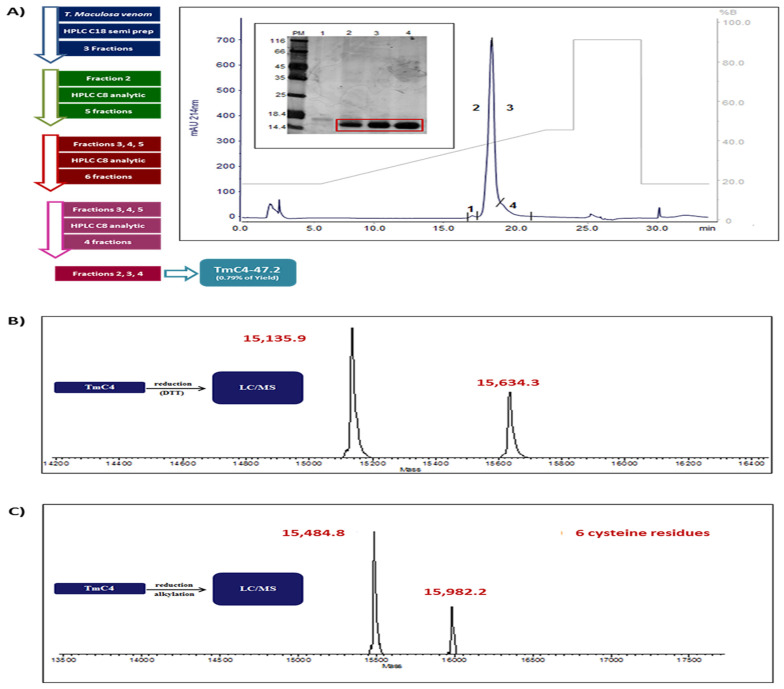
The raw venom of *Thalassophryne maculosa* was submitted to a sequence of fractionation in a high-pressure liquid chromatography (HPLC) system using a C18 followed by C8 reversed phase HPLC columns, according to the scheme in (**A**) (top left). The gradient applied was 20–80% of buffer B in 35 min, in a 1 mL min^−1^ flux. The absorbance was measured at 214 nm and the last pooled fractions containing the isolated toxin are presented in the chromatogram and highlighted in the 12% SDS-PAGE gel with a molecular mass of 15 kDa. 10 µL of the toxin collected from the last 3 fractions were applied to an Liquid Chromatography Mass Spectrometry (LC/MS) system by direct infusion to determine the mass and purity (**B**). The number of cysteines in each protein was determined by LC/MS in the reduced and alkylated samples (**C**).

These 3 pooled fractions were finally rechromatographed on C8 and generated 3 fractions with the toxin with a molecular mass of 15 kDa as shown on the 12% SDS-PAGE gel (Figure 1A). To determine the exact mass and purity of the toxin obtained, we collected the 3 fractions and applied 10 µL of the toxin to an LC/MS system by direct infusion and the deconvolution of the mass spectrum revealed two distinct proteins with very close molecular masses, one with 15,135.9 and the other with 15,634.3 Da, as observed in Figure 1B. Next, the number of cysteines in each protein was determined in the reduced and alkylated samples, a process that adds 57 Da to each cysteine residue present in the native proteins. We can observe in Figure 1C that the treatment generated proteins with masses of 15,484.8 and 15,982.8. By the difference of the masses of the pure samples and the reduced/alkylated samples, we prove the presence of 6 cysteine residues in each of the proteins according to Yang, Liu, and Liu [18].

### 2.2. TmC4-47.2 Is a Galactose-Binding Nattectin-Like Lectin

**Figure 2:**
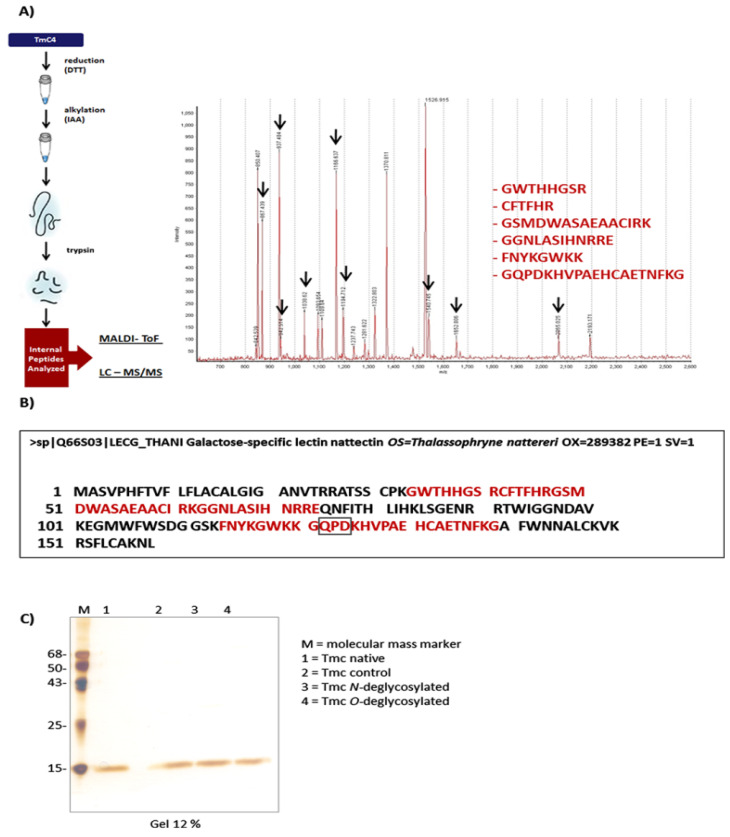
The last pool of the fractions containing the toxin obtained in the C8 column chromatography was used for mass determination and identification of internal peptides by MALDI-ToF spectrometry (**A**). The found peptides were sequenced and overlapped to the *Thalassophryne nattereri* Nattectin (GenBank LECG_THANI Galactose-specific lectin Q66S03) (**B**). Enzymatic deglycosylation was tested to check the effect on the electrophoretic mobility of the native protein in a 12% gel (**C**).

Subsequently, the pool of the 3 fractions containing the toxin obtained after the last C8 column chromatography was also used for mass determination by MALDI-ToF spectrometry. After digestion, we found 9 peptides with masses ranging from 867.439 to 2065.025 (Figure 2A), which were sequenced and compared to the sequence of Nattectin present in the venom of the fish *T. nattereri* (GenBank LECG_THANI Galactose-specific lectin Q66S03). In Figure 2B, in red, we can observe the sequences of the internal peptides overlapping in the Nattectin sequence with 100% homology and the conserved galactose-binding domain QPD (Gln-Pro-Asp) [19].

The glycan structure of nattectin-like lectin was further studied by enzymatic deglycosylation [20]. We confirmed that treatment with O-glycosidase or N-glycosidase did not alter the electrophoretic mobility of the proteins compared to the native protein, which shows a single band of 15 kDa (Figure 2C).

**Figure 3:**
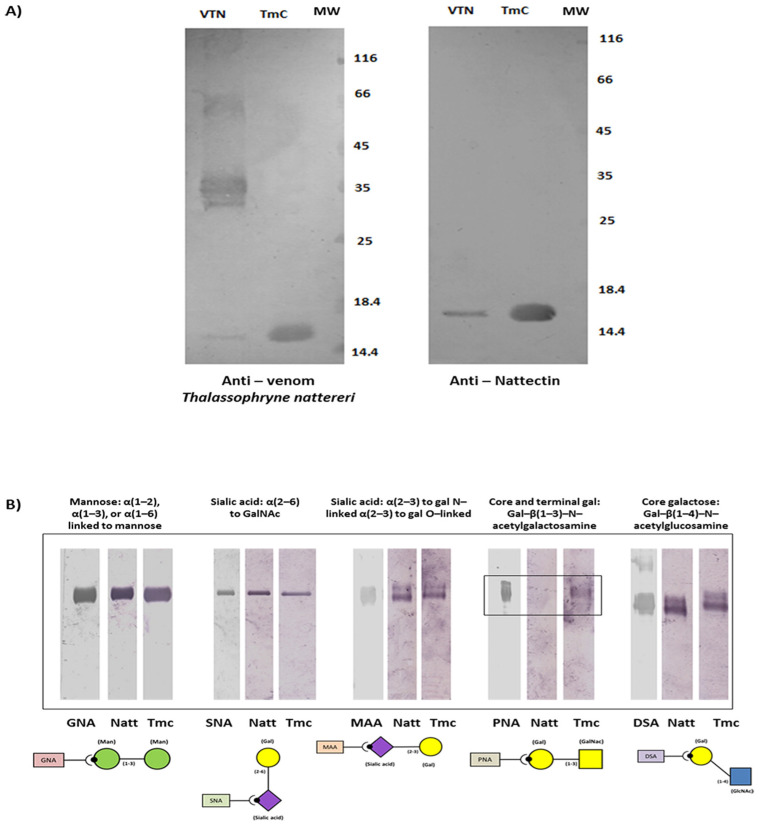
The nattectin-like toxin from *Thalassophryne maculosa* is specifically recognized by Anti-venom and Anti-Natectitn antibodies from *Thalassophryne nattereri*. Serum from mice immunized with *T. nattereri* venom-VTN or Nattectin were tested to check their ability in recognizing *T. maculosa* toxin-TmC (**A**). 10 µg of nattectin-like toxin and *T. nattereri* venom were subjected to 12% SDS-PAGE gel and transferred to a nitrocellulose membrane. The membrane was incubated with *T. nattereri* anti-Natectin anti-venom serum. They were subsequently incubated with peroxidase-labeled mouse anti-IgG and revealed with 4-α-chloro-naphtol. A digoxigenin (DIG) glycan differentiation competition assay kit (Roche Applied Science, Germany) was used to identify the binding specificity of the toxin. Toxin/carbohydrate binding was revealed by incubation with different DIG-labeled lectins and alkaline phosphatase-conjugated anti-DIG antibody (**B**).

After identifying the sequences similarities of the internal peptides of the toxin to those of Nattectin from *T. nattereri*, we confirmed the ability of serum from mice immunized with *T. nattereri* venom or Nattectin to recognize *T. maculosa* toxin (Figure 3A) as the recognition of Nattectin itself from *T. nattereri* venom, which indicates that it is a nattectin-like protein. The high affinity and narrow specificity of the nattectin-like lectin of *T. maculosa* for defined oligosaccharide structures were evaluated using the digoxigenin (DIG) Glycan Differentiation Kit, a competition assay (Figure 3B). The toxin/carbohydrate binding revealed by incubation with different DIG-labeled lectins demonstrated a weak interaction of both Nattectin and nattectin-like protein of *T. maculosa* with PNA, indicative that both Nattectin proteins recognized Gal-β(1–3)-N-acetylgalactosamine, which forms the core 1 structure of many O-glycans [21]. 

### 2.3. Hemagglutinating and Antimicrobial Activities of T. maculosa Nattectin-Like Toxin

**Figure 4:**
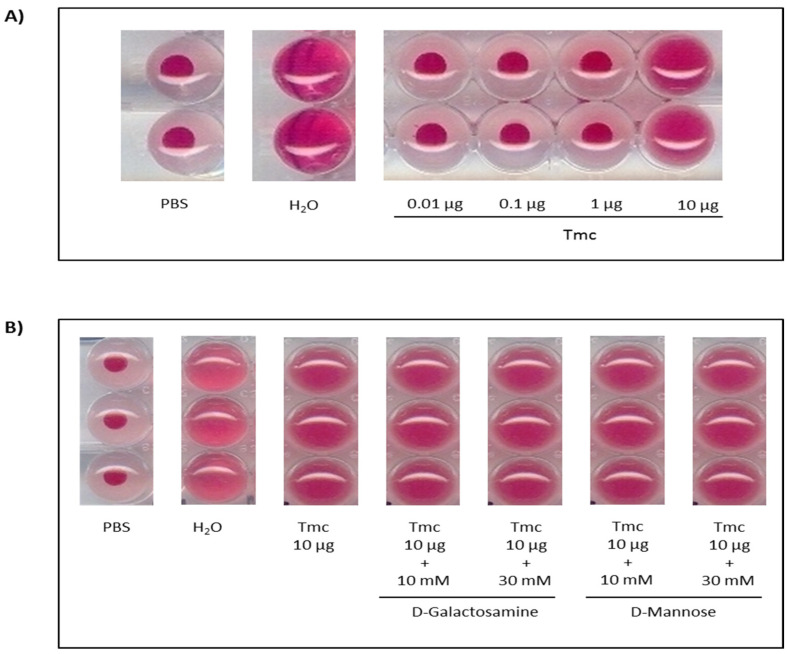
Assessment of the hemagglutinating activity of *Thalassophryne maculosa* toxin-TmC on human erythrocytes (**A**). TmC pre-incubation with D-galactosamine or D-Mannose was tested to confirm the permanence of the hemagglutinating pattern (**B**). Negative and positive controls were made by the respective addition of phosphate-buffered saline—PBS and distilled water—H_2_O.

To confirm the lectin activity of the nattectin-like protein, hemagglutinating activity was evaluated (Figure 4). Nattectin-like protein agglutinated A type-tested human erythrocytes at a dose of 10 µg. Moreover, when this dose of nattectin-like toxin was previously incubated with D-galactosamine or D-Mannose, the agglutination capacity of erythrocytes by nattectin-like was preserved.

Antimicrobial activity of nattectin-like toxin was performed against *Micrococcus luteus* A270, *Escherichia coli* SBS 363, and *Candida albicans* strains and the Nattectin from *T. nattereri* was tested in parallel as an intern control. We found that 10 µg of nattectin-like toxin did not inhibit the growth of the Gram-negative bacteria (*E. coli*) tested. Furthermore, corroborating the Nattectin effect (that inhibited the growth of all three strains evaluated at 10 µg), this dose of nattectin-like lectin showed an inhibitory effect on *M. luteus* and *C. albicans*.

### 2.4. TmC4-47.2 Toxin-Induced Alterations in the Microcirculation

**Figure 5:**
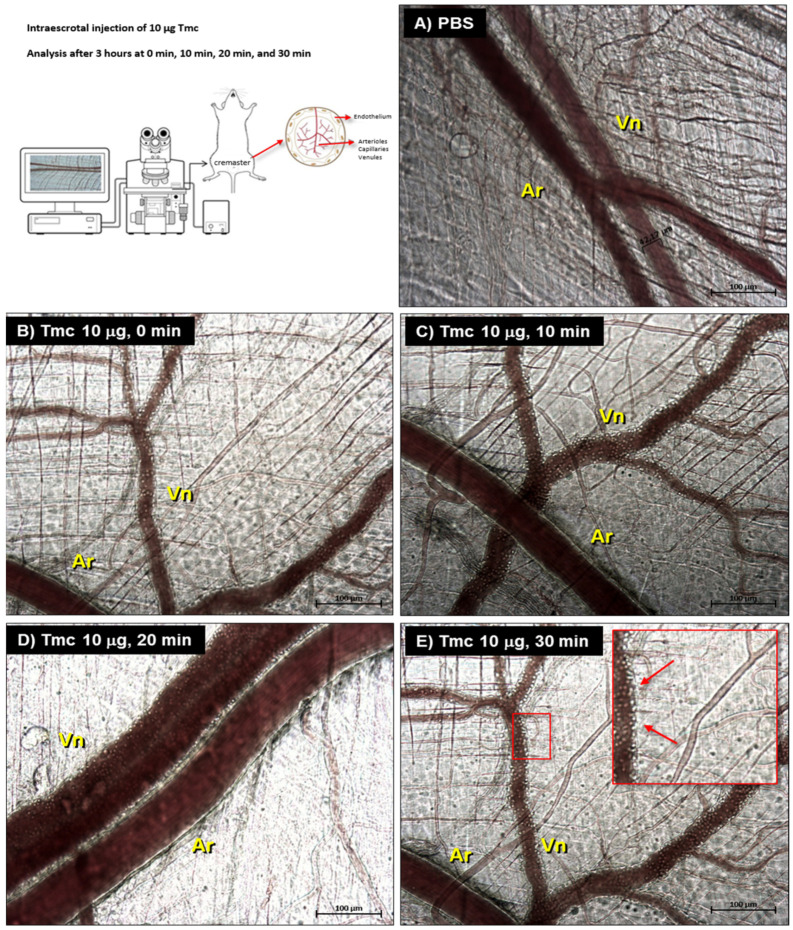
Evaluation of changes in the cremaster muscle microcirculation by intravital microscopy after 3 h of the application of 10 µg of TmC4-47.2 toxin, according to the summarized protocol illustrated in the top-left corner. The tissue microvasculature was evaluated by an optical microscope coupled to a photographic camera in the control group treated with PBS (**A**) and in the set times of 0, 10, 20, and 30 min after the 3 h of exposure (**B**–**E**). An intense migration and rolling of leukocytes have been observed. In the 6E inset, arrows evidence the leukocytes in venules. Ar: arterioles; Vn: venules.

The ability of nattectin-like toxin to induce changes in the microcirculation was evaluated using intravital microscopy assay in cremaster muscle of mice using the intra-scrotal application of 10 µg of the toxin and evaluation after 3 h of the injection. We observed in Figure 5 an intense leukocyte recruitment and rolling in the post-capillary venules immediately after the 3 h rest period (0 min.) that increased with time or stayed intense up to 30 min, as it can see from Figure 5B–E. Additionally, we registered a decrease in vessel flow after 10 min, followed by a complete stop of flow in venules and arterioles, possibly due to fibrin thrombus formation after 20 min. Nattectin-like lectin did not induce changes in the caliber of arterioles or damage to muscle fibers.

**Figure 6:**
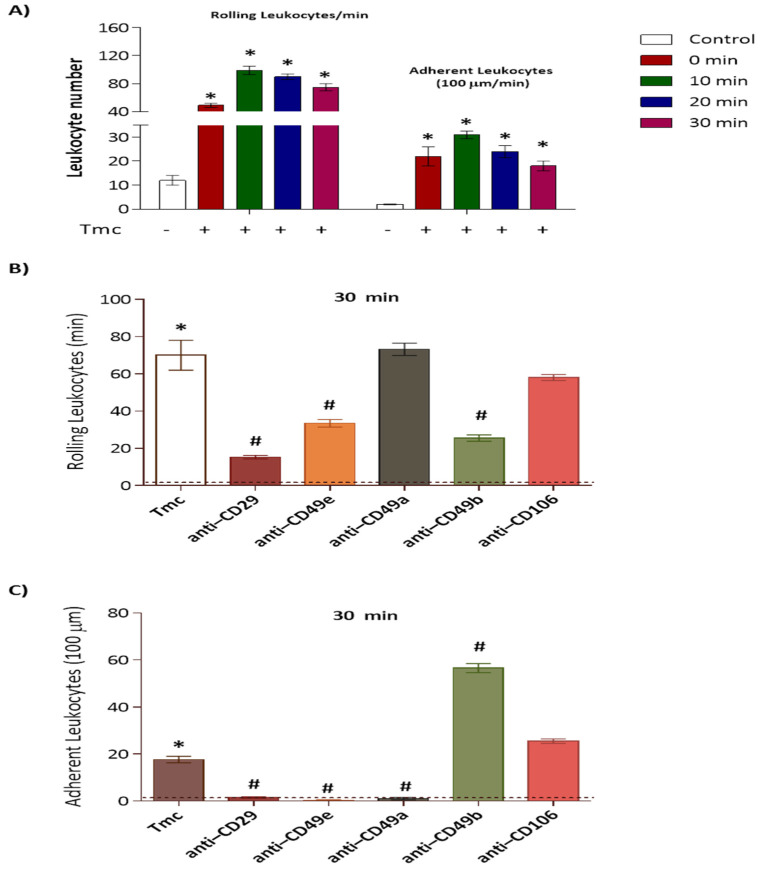
The number of rolling and adherent leukocytes controlled by 10 µg of the nattectin-like toxin TmC4-47.2 (Tmc) were counted in the post-capillary venules of mice at 0 to 30 min after 3 h of exposure using bright field intravital microscopy (**A**). The process of leukocyte recruitment, rolling (**B**), and adherence (**C**) was visualized in the cremaster vasculature of mice with inhibited alpha and beta integrins by neutralizing antibodies before intrascrotal toxin injection. The “*” represents a statistically significant difference with negative control (non-treated) represented by the dashed basal line (**B**,**C**), and the “#” represents statistically significant difference of integrins-treated groups with the *Thalassophryne maculosa* toxins (Tmc), *p* < 0.05.

Our results presented in Figure 6A demonstrate that the intense and prolonged rolling of leukocytes induced by the nattectin-like toxin was followed by adhesion in the post-capillary venules, indicating the toxin’s ability to promote extravasation of leukocytes into the surrounded interstitial tissue [22].

Integrins are a family of ubiquitous αβ heterodimeric receptors which combine with various ligands in physiological processes and disease, playing a crucial role in cell proliferation, tissue repair, inflammation, infection, and angiogenesis [23]. Next, using bright field intravital microscopy, we visualized the process of leukocyte recruitment in the cremaster vasculature of mice with alpha and beta integrins inhibited by neutralizing antibodies before intra-scrotal toxin injection. We found that treatment of mice with anti-CD29 (beta 1 integrin), anti-CD49e (alpha 5 integrin), and anti-CD49b (alpha 2 integrin) blocked 83%, 57%, and 69%, respectively, of the rolling leukocytes compared to untreated mice (Figure 6B). No inhibition was induced in mice pre-treated with anti-CD49a (alpha 1 integrin) or anti-CD106 (VCAM-1) neutralizing Abs (Figure 6B). Furthermore, the adherent leukocytes induced by nattectin-like lectin were entirely inhibited by anti-CD29, anti-CD49e, and anti-CD49a neutralizing Abs (Figure 6C). In contrast, anti-CD49b or anti-CD106 did not inhibit the adherence of leukocytes to venules.

### 2.5. Induction of Acute Inflammation by Nattectin-Like Protein

We used a mouse model of peritonitis to evaluate the inflammatory response profile induced by the nattectin-like toxin TmC4-47.2. Balb/c mice received 10 µg of the toxin intraperitoneally diluted in 500 µL of sterile phosphate-buffered saline (PBS), and control mice received only sterile PBS. Six, 16, and 24 h after the exposure, the animals were sacrificed, and the peritoneal cavity was washed to obtain the cell suspension. We analyzed the leukocyte influx recruited to the peritoneum by labeling surface molecules typical for each cell population and the dosage of cytokines (IL-1β, IL-6, and TNF-α) and chemokines (MCP-1, KC, and eotaxin) involved in the inflammatory process.

**Figure 7:**
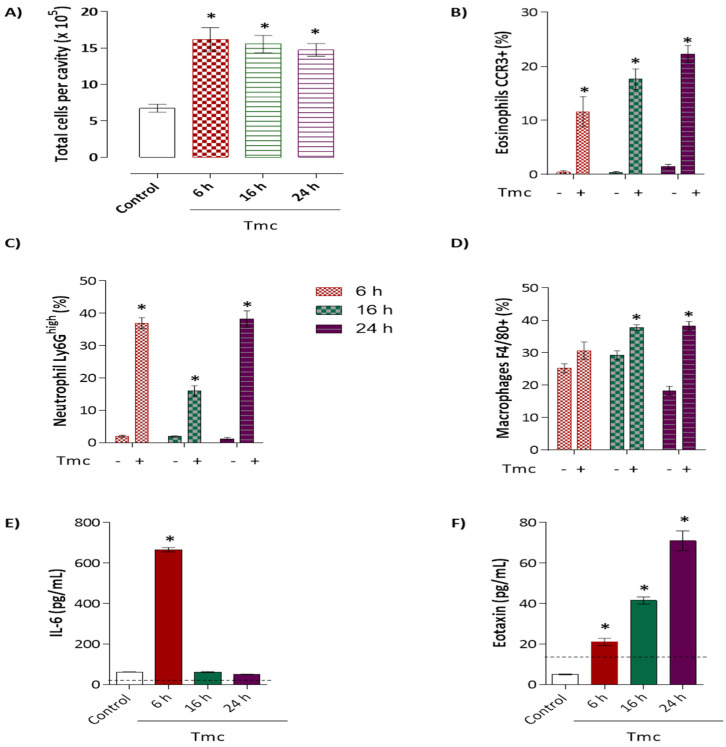
The inflammatory response profile induced by the *Thalassophryne maculosa* toxin TmC4-47.2 (Tmc) was evaluated in a Balb/c mice model of peritonitis. The toxin was applied intraperitoneally at 10 µg in 500 µL of sterile phosphate-buffered saline (PBS). Control mice received sterile PBS. At six, 16, and 24 h after the exposure, the cell suspension from the peritoneal cavity was collected. The total cell number (**A**), the leukocyte influx to the peritoneum (**B**–**D**), and the dosage of IL-6 (**E**) and eotaxin (**F**) involved in the inflammatory process was analyzed.The “*” represents a statistically significant difference with negative control (non-treated), *p* < 0.05.

Our results in Figure 7A show that mice injected with the nattectin-like toxin exhibited intense leukocyte extravasation into the peritoneal cavity after 6 h of injection that was persistent for 24 h. The acute phase (6 h) of inflammation was characterized by the influx of eosinophils (Figure 7B) and mainly neutrophils (Figure 7C). After 16 h, macrophages (Figure 7D) entered the inflamed peritoneal cavity and remained for 24 h in the presence of a large number of both granulocytes.

Finally, we observed the production of IL-6, an important cytokine involved in the inflammatory process, only within 6 h after the 10 µg toxin injection (Figure 7E). Eotaxin, a chemotactic for eosinophils, was produced 6 h after injection, and increasing levels were observed up to 24 h (Figure 7F). In contrast, the injection did not promote the secretion of the cytokines IL-1β and TNF-α, as well as the neutrophil chemotactic factor, KC (data not shown).

## 3. Discussion

The *T. nattereri* envenomation in humans and the recapitulation of the injury in mice have been extensively studied by our group [24,25,26]. We identified in the Natterin group of venom toxins the aerolysin proteins being responsible for the main effects of envenomation [27,28], and their immunopharmacological activities were determined [29,30,31,32]. Besides the Natterin proteins, *T. nattereri* venom also contains Nattectin, a lectin with Ca^2+^-independent hemagglutinating and immunomodulatory activities [4]. Nattectin shows the particular potential to bind types I and V collagen and improve integrin-mediated HeLa cell adhesion and apoptosis protection by its binding to RGD-dependent integrins, mainly the β1 subunit [33]. We further demonstrated its ability to activate antigen-presenting cells [34] and trigger Th1-type immune response with IgG1 production [35].

On the other hand, just a few studies have been conducted with *T. maculosa*, one of the *T. nattereri*’s closest relatives. Sosa-Rosales et al. [17] partly identified a biologically active protein by chromatography. To obtain the toxin TmC4-47.2 from the venom of the *T. maculosa*, we used reverse-phase HPLC-based workflow with the application of the crude venom to the chromatography system coupled to a semi-preparative reverse-phase C18 column, followed by subsequent chromatography of the fractions containing the toxin with a molecular weight of 15 kDa on a reverse-phase C8 analytical column. Finally, the protein was purified into three lyophilized fractions for verification of purity and exact mass by LC/MS. Interestingly we confirmed the presence of two proteins with very close molecular weights, one with a calculated molecular weight of 15,135.9 Da and the other with 15,634.3 Da, unlike the migrated single band ~15 kDa via reducing SDS-PAGE.

To determine the number of cysteine residues and, consequently, of disulfide bridges, we proceeded with the reduction and alkylation of the toxins. After applying the sample to the same LC/MS system, we once more detected the presence of two proteins, now with 15,484.8 Da and 15,982.8 Da, respectively. From the difference in the masses of the pure proteins, we inferred that both proteins have 6 cysteines each and consequently form 3 disulfide bridges, as described in the sequence of Nattectin from *T. nattereri* [28]. Our findings that the anti-venom serum and the anti-Nattectin serum from *T. nattereri* recognize the TmC4-47.2 toxin from *T. maculosa* support the similarity of the sequences.

The sequencing of internal fragments of the toxins generated by enzymatic digestion with trypsin and analysis by MALDI-ToF revealed 100% similarity of the sequenced fragments with the Nattectin sequence, which correspond to 40% of its entire sequence. The high homology rate might reflect a relatively small evolutionary divergence between the two congeneric species and the fact that they harbor the same biological properties. Batrachoididae, the only family in the ray-finned fish order Batrachoidiformes, arose on Earth in the Miocene, which is the first geological epoch of the Neogene Period that extends from about 23 to 5 million years ago (mya). There are about 83 species of toadfishes grouped into 21 genera. The subfamily Thalassophryninae has six phenotypically alike species in the genus *Thalassophryne*, which suggests the time of divergence among species within this genus must be little, although there is no precise description of the time of divergence in the literature. Using peptide mass fingerprinting, we identified a unique carbohydrate recognition domain composed of the amino acids glutamine, proline, and aspartate (QPD), a specific galactose-binding site [36]. We have designated the TmC4-47.2 toxin as a galactose-binding protein.

Glycan structures are common post-translational modifications of proteins, assisting in protein folding and defining the timing for protein disposal as a part of the quality control function in the early secretory pathway [37]. Our data shows that the nattectin-like protein of *T. maculosa* is non-glycosylated since we determined the absence of GlcNAc-β-Ser/Thr or N-linked glycan residues.

Among the distinct post-translational modifications, glycosylation is the most recurrent, and virtually 50% of all known proteins are thought to be glycosylated. In contrast, the galectins are generally small, soluble, non-glycosylated proteins and, unlike the C-Type lectins, do not require structural stabilization via Ca^2+^ complexing for their activity. However, many C-type lectins are Ca^2+^-independent and may not necessarily bind to sugar ligands [38]. Our findings showing the nattectin-like lectin as a non-glycosylated protein with the ability to interact with the core and terminal galactose-type ligands point to a functional similarity with proto-type galectins that contains only one CRD. Galectins, a family of animal lectins with an affinity for β-galactosides, can form multivalent complexes with cell surface glycoconjugates and trigger several intracellular signals to modulate cell activation, differentiation, and survival [39].

Literature has portrayed the protective role of galectins against infections when binding to the glycoconjugates on the surface of invasive microbes (virus, bacteria, fungi, and parasites) acting as pattern recognition receptors in innate immunity [40]. In fish, they have also been demonstrated to participate in pathogen recognition. Gal-1, a proto-type galectin, from rock bream (*Oplegnathus fasciatus*) bound and agglutinate many bacteria [41] and the Gal-1 of zebrafish bound infectious hematopoietic necrosis virus (IHNV) in a carbohydrate-dependent way [42]. AJL-1, a proto-type galectin of Japanese eel (*Anguilla japonica*), was reported to hinder the biofilm formation of *Aggregatibacter actinomycetemcomitans* [43]. The galectin-8 and -9 of mandarin fish (*Siniperca chuatsi*) were shown to inhibit the growth of some pathogens [44]. Our results corroborate these findings, and through functional analogies we confirmed the ability of nattectin-like protein to induce carbohydrate-independent agglutination of human erythrocytes and exert antimicrobial activity against gram-negative and gram-positive bacterial pathogens as well as against *C. albicans*.

A substantial number of lectins have been identified in other fish. Notably the galectins, which is unusual compared to venom from other animals, like snakes, that commonly present C-type lectins [45]. Probably due to the complex environment, fish have evolved natural immune modulators, such as galectins and C-type lectins. In the fish lectins, evolution likely led to the acquisition of galectin-like properties. Thus, the functional similarities between Nattectins and Galectins studied herein might be the result of convergent evolution and because Nattectin belongs, from an evolutionary and structural perspective, to the C-type lectin family. This is also corroborated by the similarity of *T. maculosa*’s and *T. nattereri*’s Nattectin that present highly conserved residues of carbohydrate-recognition domains found in the C-type lectins.

In addition to the role of galectins in microbial death, several reports show their importance in the transendothelial migration of neutrophils and eosinophils [46,47]. Intravital imaging has yielded relevant understandings into the regulation of inflammatory responses to pathogens and sterile insults in a variety of tissues [48]. Such imaging approaches were useful in characterizing individual steps of the leukocyte recruitment cascade. Moreover, our data show that nattectin-like lectin modulates leukocyte rolling and adherence to vascular endothelium dependent on α5β1 integrin. This crucial adhesion molecule mediates the adherence of many cell types to the extracellular matrix by recognizing its classic ligand fibronectin [49]. It has been reported that the increased expression of α5β1 integrin and fibronectin enrichment in the epidermis are seemingly features of chronic neutrophilic inflammation [50]. Furthermore, polymorphonuclear neutrophils (PMNs) express several surface integrins, including α5β1 [51].

Additionally, we noted that nattectin-like toxin increased eosinophil recruitment to the peritoneal cavity of mice and the production of the pro-inflammatory chemokine, eotaxin. The trafficking of primed mature eosinophils from the bloodstream into inflamed tissues is finely regulated by adhesion molecules, several cytokines, and chemokines with overlapping functions. Studies with mouse and human eosinophils have shown that rolling along the vascular endothelium and the activation-dependent stable adhesion to the vascular endothelium are supported by β1 integrins. Trans-endothelial migration is under the control of eotaxin and its receptor, CCR3 [52].

Together, our findings show that *T. maculosa* possesses two non-glycosylated nattectin-like proteins with similar galactose-binding specificity. Nattectin-like lectin retained their ability to induce carbohydrate-independent agglutination of human erythrocytes. Furthermore, it exerted potent antimicrobial activity against gram-negative and gram-positive bacterial pathogens, as well *C. albicans*. The non-glycosylation status of nattectin-like proteins did not impact protein function since lectins showed a substantial effect on leukocytes rolling and adherence to the endothelial wall of the mice’s cremaster muscle. This interaction was dependent on β1 integrin binding. The inflammation of the peritoneal cavity was characterized by eosinophils and neutrophils influx recruited in response to IL-6 and eotaxin mediators produced by nattectin-like protein. Our results point to an immune function of nattectin-like toxin participating in the antimicrobial response through the recognition of pathogens via carbohydrate-binding and modulation of eosinophil and neutrophil infiltration in inflammation.

## 4. Materials and Methods

### 4.1. Thalassophryne maculosa Venom and Isolation of TmC4-47.2 Toxin

All necessary permits for the capture of *T. maculosa* and to collect the venom were obtained from the Escuela de Ciencias Aplicadas del Mar, Núcleo Nueva Esparta, Universidad de Oriente, Isla Margarita; Venezuela (Permit Number: 30/07). *T. maculosa* individuals were transported to the laboratory and were anesthetized with 2-phenoxyethanol before sacrifice. Venom was shortly obtained from the tip of the spines by applying pressure at their bases. After centrifugation, venom was pooled and stored at −80 °C before use. Protein content was examined using bovine serum albumin (Sigma Chemical Co., St Louis, MO, USA) as standard. Endotoxin content was evaluated (resulting in a total dose < 0.8 pg LPS) with QCL-1000 chromogenic Limulus amoebocyte lysate assay (Bio-Whittaker) according to the manufacturer’s instructions. Aliquots of crude venom from *T. maculosa* (5 mg mL^−1^) was submitted to a reversed-phase (RP) LC10A high-performance liquid chromatography system (HPLC) Shimadzu (Äkta, Amersham Biosciences, Sweden) starting in a C18 semi-preparative column (Shimadzu-Shim Pack-CLC-ODS: 4.6 mm ID × 25 cm, 5 µm, and 100 Å). For elution, a binary gradient of 0.1% TFA aqueous solution (A) and 0.1% TFA 90% acetonitrile solution (B) was used, with a flow rate of 5.0 mL/min for 60 min, and detection at 214 nm absorbance. The chromatographic fractions were manually collected, vacuum dried, and stored at −20 °C. All fractions containing the toxin were identified by 12% SDS-PAGE [53]. The chromatographic fraction containing the toxin was submitted to 3 chromatographic steps using a Jupiter C8 4.60 mm × 250 mm, 10 µm, 300 Å, column (Phenomenex). The elution method was 20% to 80% B solution from 0 to 35 min at 1 mL/min, followed by a gradient of 10% B solution per minute until reaching a maximum concentration of 50% acetonitrile. The presence of the toxin in the eluates was confirmed by absorbance and SDS-PAGE.

### 4.2. TmC4-47.2 Toxin Mass Determination

To determine the toxin mass, we used 10 μL of the sample injected into an LC-MS Surveyor MSQ Plus system (Thermo Finnigan) by direct infusion in a flow of 50 μL/min of aqueous acetonitrile solution (1:1) containing 0.1% formic acid under capillary voltage conditions 3.1 kV, 75 V cone, scan lasting 1 s in the *m*/*z* range 200–2000. The equipment was previously calibrated with sodium iodide (*m*/*z* range 22.98 to 1971.61). To obtain the molecular weight of the sample, the deconvolution of the mass spectrum obtained was performed using the Mag Tran program version 1.0 beta 8 [54]. For MALDI-ToF mass spectrometry, analyzes were performed in a MALDI-ToF/PRO instrument (Amersham). The samples in solution were mixed 1:1 (*v*/*v*) with a supersaturated matrix solution for proteins (sinapic acid), deposited on the sampling plate (0.4–0.8 μL), and allowed to dry at room temperature. The spectrometer was operated in reflection mode, and P14R ([M+H+]^+^ 1533.85) and angiotensin II ([M+H+]^+^ 1046.54) (Sigma, St. Louis, MO, USA) were used as external calibrants.

### 4.3. Reduction, Alkylation, and Digestion of TmC4-47.2 Toxin

The purified toxin was used for the identification of disulfide bridges, according to Yang, Liu, and Liu [18]. The toxin was reduced by 10 µL of 5 mM DTT in 25 mM ammonium bicarbonate (Sigma-Aldrich, St. Louis, MO, USA) and heated at 60 °C for 30 min. For alkylation, 10 μL of 100 mM iodoacetamide in 25 mM ammonium bicarbonate (Sigma-Aldrich, St. Louis, MO, USA) was added and kept for 30 min in the dark. For enzymatic digestion, 2 μL of ultra-grade trypsin (Proteomics grade; Sigma, St. Louis, MO, USA) solution at 40 ng·μL^−1^ was added to 8 μL of the toxin solution and incubated 18 h at 37 °C. The samples were analyzed using a nanoflow LC/MS/MS system customized with a PepFinder Kit. Aliquots of 10 μL were initially charged onto a reversed-phase peptide trap column in a of 10 μL/min flow rate for 3 min. Then peptides were eluted and partitioned on a reversed-phase capillary column (PicoFritTM; 5 μm BioBasic^®^ C18, 300 Å pore size; 75 μm × 10 cm; tip 15 μm, New Objective, Woburn, MA, USA). Solution A comprised 0.1% formic acid, and Solution B acetonitrile and 0.1% formic acid. The flow rate began at 100% A at 10 μL/min for 3 min. Next, it was raised to 70 μL/min for 6.9 min at 100% A and the gradient commenced at 100% A and 0% B. The gradient was increased to 50% B in 60 min, then to 90% B in 5 min and later diminished to 0% B in 5 min and sustained at 100% A for 10 min. The complete program duration was 110 min. By the PepFinder Kit, the flow was separated in a 1:100 ratio. Hence, the factual flow rate injected into the mass spectrometer was 0.5 μL/min. The HPLC was coupled to a Finnigan LCQ Deca XP Plus ion trap mass spectrometer supplied with a nanospray ionization font. Spray voltage was established at 2.5 kV, and the equipment was managed in a data-dependent method, in which one MS scan was captured in the 300−1600 m/z range followed by MS/MS acquisition using collision-induced segregation of the 10 most intense ions from the MS scan. Dynamic peak exclusion was used to avoid the same m/z being chosen for the following 120 s. Tandem mass spectra were extracted by Xcalibur software (version 2.0; Thermo scientific, Waltham, MA). The subsequent MS/MS spectra were searched through a MASCOT search engine (Matrix Science, London, UK) against the NCBI NR database in the taxa Chordata with a 1.20 Da parent tolerance and 0.60 Da fragment tolerance. Iodoacetamide derivatives of cysteine and oxidation of methionine were detailed in MASCOT as fixed and variable modifications, respectively. Scaffold (version Scaffold_2_04_00, Proteome Software Inc., Portland, OR) was used to confirm MS/MS-based peptide and protein description. Peptide distinguishing were credited if they exceeded certain database search engine thresholds. MASCOT descriptions required ion scores higher than the associated identity scores and 20, 30, 40, and 40 for singly, doubly, triply, and quadruply charged peptides. X! Tandem identifications needed at least –Log (Expect Scores) scores of greater than 2.0. Protein identifications were considered if they had at least 2 identified peptides.

### 4.4. Enzymatic Deglycosylation

In denaturing conditions, for protein deglycosylation, toxin (3.4 μg) were set in 20% SDS for 1 min at 95 °C. After adding 0.2 M sodium phosphate buffer, 0.08% sodium azide, 0.5 M EDTA pH 8, 10% nonidet P-40, incubation was prolonged for 2 min at 95 °C. After cooling, 1 U.μL^−1^ of N-glycosidase F (Roche, Mannheim, Germany) or 25 mU.50 μL^−1^ O-glycosidase was added, and the mixture was incubated for 18 h at 37 °C. The deglycosylation profiles were evaluated by SDS-PAGE.

### 4.5. Immunoblotting

The purified protein (10 μg) of *T. maculosa* venom was analyzed by 12% SDS-PAGE under non-reducing conditions. Venom from *T. nattereri* (V*Tn*) was used as a control. After SDS-PAGE and transfer to nitrocellulose membrane (pore size = 0.2 µm, Schleicher and Schüll, Dassel, Germany), the toxin was detected using plasma from mice sensitized with *T. nattereri* venom (1:20 dilution) or from Nattectin-sensitized mice followed by Goat anti-mouse IgG HRP (sc-2005 Santa Cruz, at 1:2000) as a secondary antibody (Ab).

### 4.6. Determination of TmC4-47.2 Toxin Binding Specificity to Carbohydrates

The toxin was cut from 12% SDS-PAGE gel and eluted in PBS to identify toxin binding specificity using a competition assay performed according to Haab [55] using the DIG Glycan Differentiation Kit (#11210238001, Roche Applied Science, Germany). The pure control glycoproteins: carboxypeptidase Y, transferrin, fetuin, and asialofetuin or after pre-incubated with 1 µg of the toxin TmC4-47.2 for 1 h at 37 °C were administered in a 12% SDS–PAGE gel and transferred to a nitrocellulose membrane. Toxin/carbohydrate binding was revealed by incubation with different DIG-labeled lectins and alkaline phosphatase-conjugated anti-DIG antibodies (Roche Molecular Biosciences). Characteristics of specific binding of lectins to carbohydrate moieties used to identify these structures in this study: GNA: *Galanthus nivalis* agglutinin specific to mannose: α(1–2), α(1–3), or α(1–6) linked to mannose; SNA: *Sambucus nigra* agglutinin specific to Sialic acid: α(2–6) to GalNAc; MAA: *Maackia amurensis* agglutinin specific to Sialic acid: α(2–3) to galactose N-linked α(2–3) to galactose O-linked; PNA: *Arachis hypogaea* Peanut agglutinin specific to Core and terminal galactose: Gal-β(1–3)-N-acetylgalactosamine; and DSA: *Datura stramonium* agglutinin specific to Core galactose: Gal-β(1–4)-N-acetylglucosamine.

### 4.7. Hemagglutinating and Antimicrobial Activities

Human erythrocytes (type A) were collected in 0.15 M citrate buffer, pH 7.4, and washed 3 times by centrifugation with 0.15 M PBS, pH 7.4. To assess the hemolytic activity, aliquots of 10 µL of the selected toxin at 0.01, 0.1, 1, or 10 µg were put in 50 µL in a 3% suspension of erythrocytes in wells of U-shaped bottom plates and incubated for 3 h at room temperature. Solutions of isolated toxin at 10 µg previously incubated with D-Galactosamine (12662, Sigma) or D-Mannose (M2069, Sigma) both at 10 or 30 mM for 1 h were also usedThe hemagglutinating activity was verified by reading the absorbance at 595 nm of each well in a plate reader. Erythrocytes incubated with water were used as positive control (100% hemolysis). Antimicrobial potential was monitored by a liquid growth inhibition assay against *M. luteus* A270, *E. coli* SBS 363, and *C. albicans* MDM8, as described by Rossi et al. [56]. Pre-inoculum of the strains was prepared in Poor Broth (PB broth, 1.0 g peptone in 100 mL of H_2_O containing 86 mM NaCl at pH 7.4; 217 mOsM for *M. luteus* and *E. coli* and 1.2 g potato dextrose in 100 mL of H_2_O at pH 5.0; 79 mOsM for *C. albicans*), at 37 °C under agitation. The absorbance at 595 nm was set on, and one aliquot of this solution was taken to get cells in logarithmic growth (A_595 nm_ ~0.6) and diluted 600 times (A_595 nm_ = 0.0001). The isolated toxin or Nattectin, both at 10 μg, was dissolved in sterile Milli-Q water to 100 μL of PB broth. Tetracycline and Gomesin were used as inhibitor controls. After 18 h of incubation at 30 °C, the inhibition of bacterial growth was determined by measuring absorbance at 595 nm.

### 4.8. In Vivo Experimental Protocol for Intravital Microscopy

Initially, Balb/c mice were injected by the intrascrotal route with 50 μL of TmC4-47.2 at 10 μg and rested for 3 h. Independent groups of mice were pre-treated with 500 µL of intraperitoneal (i.p.) injection of the purified rat anti-mouse CD29 (beta 1 integrin, 14-0299-82, eBioscience), hamster anti-mouse CD49a (alpha 1 integrin, PA5-95563, Thermo Fisher eBioscience), rat anti-mouse CD49b (alpha 2 integrin, ab238665, Abcam), rat anti-mouse CD49e (alpha 5 integrin, ab221606, Abcam), and rat anti-mouse CD106—VCAM-1, 14-1061-82, Thermo Fisher eBioscience) monoclonal antibodies at 10 μg mL^−1^, 30 min before TmC4-47.2 injection. Control animals received the same amount of control isotype IgG. Negative control was injected with intrascrotal sterile PBS. The study of the microvascular system was performed with an optical microscope (Axio Imager A.1, Carl-Zeiss, Oberkochen, DE ) coupled to a photographic camera (IcC 1, Carl-Zeiss, Germany) through a 10/025 longitudinal distance objective/numerical aperture and 1.6 optovar. The surgical cremaster preparation was handled as described previously [12]. Mice were anesthetized by an i.p. injection of 2% Xylazine—(Calmiun^®^, Agener União, São Paulo, Brazil) and with 0.5 g Kg^−1^ of ketamine (Holliday-Scott SA, Buenos Aires, Argentina). The scrotum was exposed, and the cremaster muscle reached. Following the incision with cautery and spreading the muscle over a cover glass, the epididymis and testis were mobilized and pinned aside, allowing the microscopic access to the muscle microcirculation. The exposed tissue was superfused with 37 °C warmed bicarbonate-buffered salines, pH 7.4. The post-capillary venules with a diameter of 25–40 µm were chosen, and the interaction of leukocytes with the luminal surface of the venular endothelium was evaluated, counting the number of rolling leukocytes every 10 min after application of inflammatory agent for 30 min. Rolling leukocytes were defined as those moving at a velocity less than erythrocytes and demonstrated a clear rolling motion. The number of adherent cells was expressed as the number per 100 μm length of venule. The experiments were carried out under the National Council for Animal Experiment Control (CONCEA) and approved by the Butantan Institute’s Animal Use Ethics Commission (CEUAIB #275/06).

### 4.9. Acute Inflammation Induced by TmC4-47.2

Balb/c mice were intraperitoneally injected with TmC4-47.2 at 10 μg in 500 μL, according to Lima et al. [32]. As a negative control, mice were injected i.p. with PBS. After 2, 16, and 24 h, the peritoneum exudates were harvested for total and differential cell count and protein determination. IL-6, TNF-α, IL-1β, KC, eotaxin, and MCP-1 were analyzed using a specific two-site sandwich ELISA with OpEIA Kits (BD-Pharmingen, San Diego, CA, USA). The total leukocyte count was performed in Turk solution, and for differential counts, neutrophils (Ly6G^+^), eosinophils (CCR3^+^), and macrophages (F4/80^+^) were identified by flow cytometry and based on staining and morphologic characteristics using a light microscope Axio Imager A1 (Carl Zeiss, Germany) with an AxioCam ICc1 digital camera (Carl Zeiss).

### 4.10. Statistical Analysis

All values were expressed as mean ± SEM. Experiments using 3 to 5 mice per group were performed independently two times. Parametric data were evaluated using analysis of variance, followed by the Bonferroni test for multiple comparisons. Non-parametric data were assessed using the Mann-Whitney test. Differences were considered statistically significant at *p* < 0.05 using GraphPad Prism (Graph Pad Software, v6.02, 2013, La Jolla, CA, USA).

## Supplementary Materials

The following are available online at https://www.mdpi.com/article/10.3390/toxins14010002/s1. Figure S1: (A) Fractionation process of the *Thalassophryne maculosa* venom. A total of 5 mg of venom was applied to a semi-preparative reversed-phase C18 column coupled to a high-pressure liquid chromatography (HPLC) system. The gradient used was from 20 to 80% buffer B in 60 min under 5 mL/min flow rate. The absorbance was monitored at 214 nm. (B) The protein content was evaluated by 12% SDS-PAGE electrophoresis in polyacrylamide gel (10 µg/well). Samples 1, 2, and 3 correspond to the fractions obtained by chromatography. MW corresponds to molecular mass markers, and the band circled in red refers to the TmC4-47.2.; Figure S2: (A) Second fractioning step for toxin purification. Fraction 2 (1 mg mL^−1^) obtained in the first chromatography was applied to a reversed-phase analytical C8 column coupled to a high-pressure liquid chromatography (HPLC) system. The gradient used was 20 to 80% of buffer B over 35 min under a flow rate of 1 mL/min. The absorbance was monitored at 214 nm. (B) The protein content of fractions 1, 2s, 2d, 3 and 4 was evaluated by 12% SDS-PAGE electrophoresis on polyacrylamide gel (10 µg/well). MW corresponds to molecular mass markers. The bands highlighted in red refer to TmC4-47.2 toxin; Figure S3: (A) Third fractionation for purification of *Thalassophryne maculosa* TmC4-47.2 toxin. Fractions 2s, 2d and 3 were mixed and 1 mg mL^−1^ of the pool was applied to a reversed-phase analytical C8 column coupled to a high-pressure liquid chromatography (HPLC) system. The gradient used was 20 to 80% buffer B over 35 min under 1 mL min^−1^ flow rate. The absorbance was monitored at 214 nm. (B) The protein content of fractions 1, 2s and 2d was evaluated by 12% SDS-PAGE electrophoresis on polyacrylamide gel (10 µg/well). MW corresponds to molecular mass markers and the bands highlighted in red refer to TmC4-47.2 toxin.
