# Supplementary material for: Molecular Characterization and Functional Analysis of the Nattectin-like Toxin from the Venomous Fish Thalassophryne maculosa
Source: Toxins (Basel). 2021 Dec 21;14(1):2. doi: 10.3390/toxins14010002 (PMC8778695; doi:10.3390/toxins14010002)
Supplement: Supplementary file 1 [file toxins-14-00002-s001.zip › toxins-1442609-supplementary.pdf]

Supplementary Materials: Molecular characterization and functional analysis of the nattectin-like toxin from the venomous fish *Thalassophryne maculosa*

Monica Lopes-Ferreira, Ines Sosa-Rosales, Pedro Ismael Silva Junior, Katia Conceicao, Adolfo Luis Almeida Maleski, Leticia Balan-Lima, Geonildo Rodrigo Disner, Carla Lima

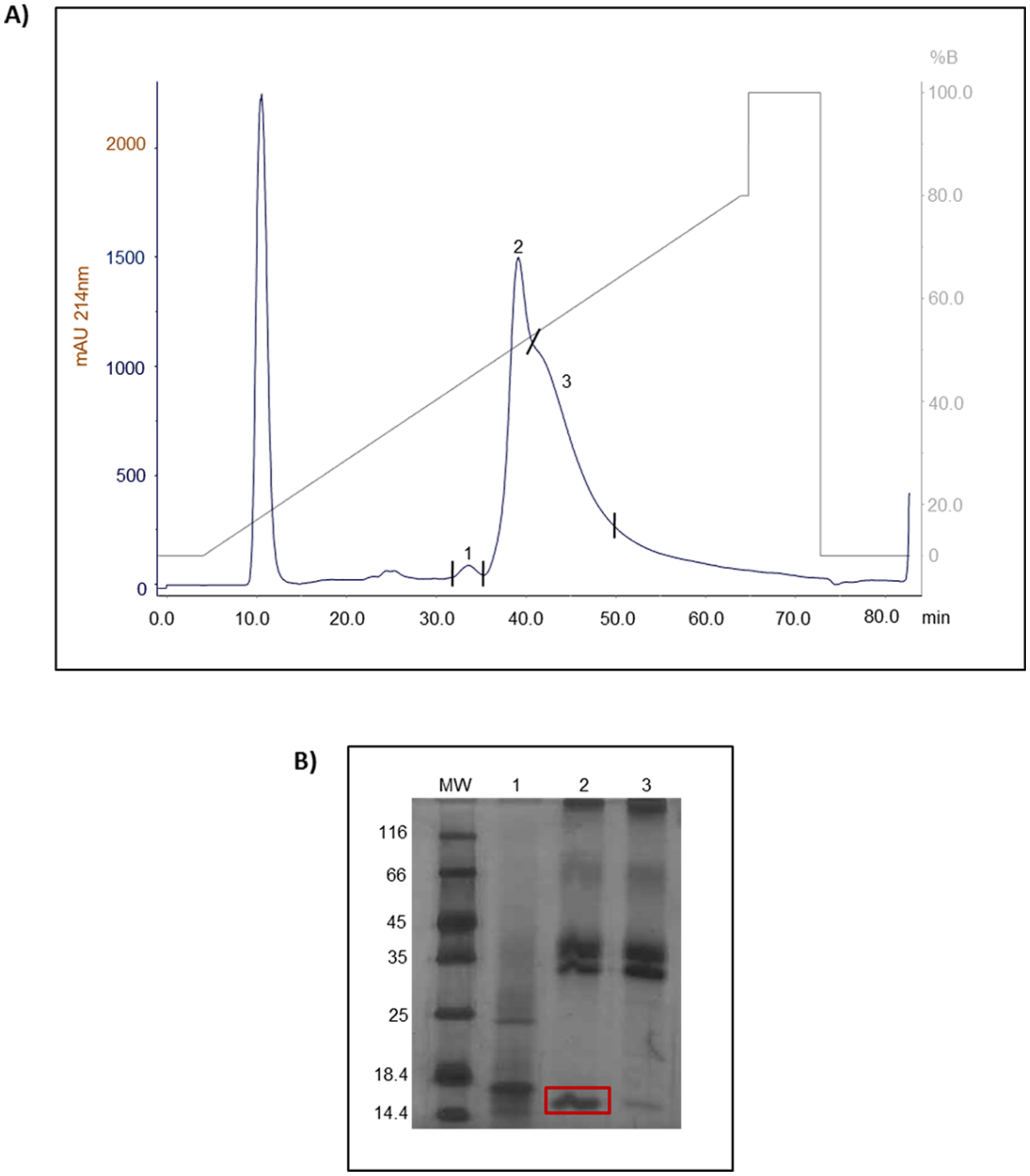

Figure S1. A) Fractionation process of the *Thalassophryne maculosa* venom. A total of 5 mg of venom was applied to a semi-preparative reversed-phase C18 column coupled to a high-pressure liquid chromatography (HPLC) system. The gradient used was from 20 to 80% buffer B in 60 min under 5 mL/min flow rate. The absorbance was monitored at 214 nm. B) The protein content was evaluated by 12% SDS-PAGE electrophoresis in polyacrylamide gel (10 µg/well). Samples 1, 2, and 3 correspond

to the fractions obtained by chromatography. MW corresponds to molecular mass markers, and the band circled in red refers to the TmC4-47.2.

A)

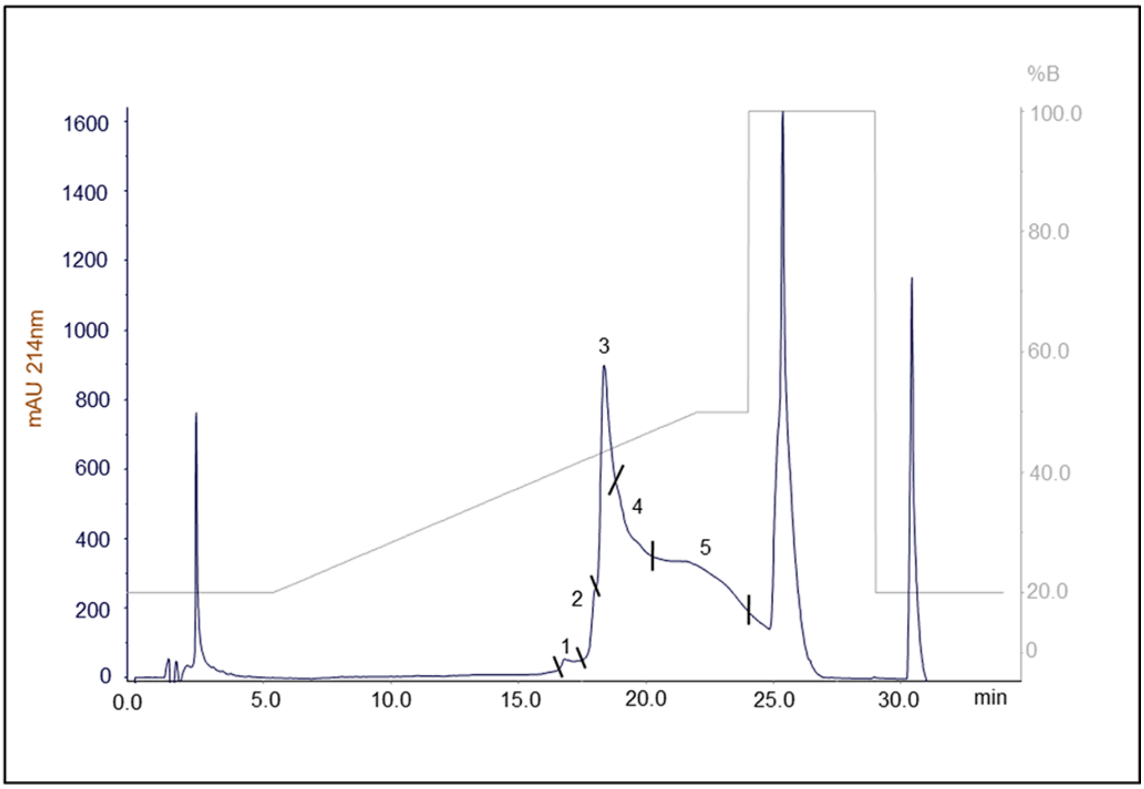

B)

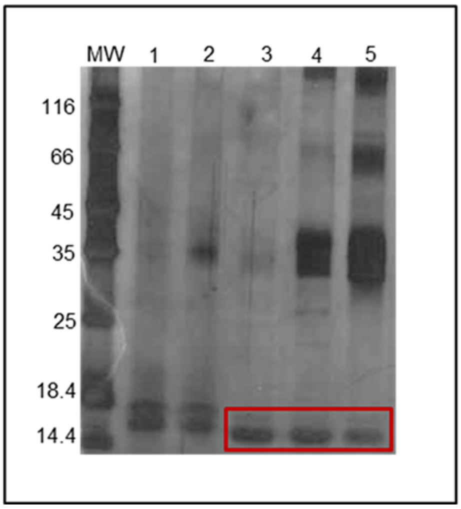

Figure S2. A) Second fractioning step for toxin purification. Fraction 2 (1 mg.mL^{-1}) obtained in the first chromatography was applied to a reversed-phase analytical C8 column coupled to a high-pressure liquid chromatography (HPLC) system. The gradient used was 20 to 80% of buffer B over 35 minutes under a flow rate of 1 mL/min. The absorbance was monitored at 214 nm. B) The protein content of fractions 1, 2s, 2d, 3 and 4 was evaluated by 12% SDS-PAGE electrophoresis on polyacrylamide gel (10 µg/well). MW corresponds to molecular mass markers. The bands highlighted in red refer to TmC4-47.2 toxin.

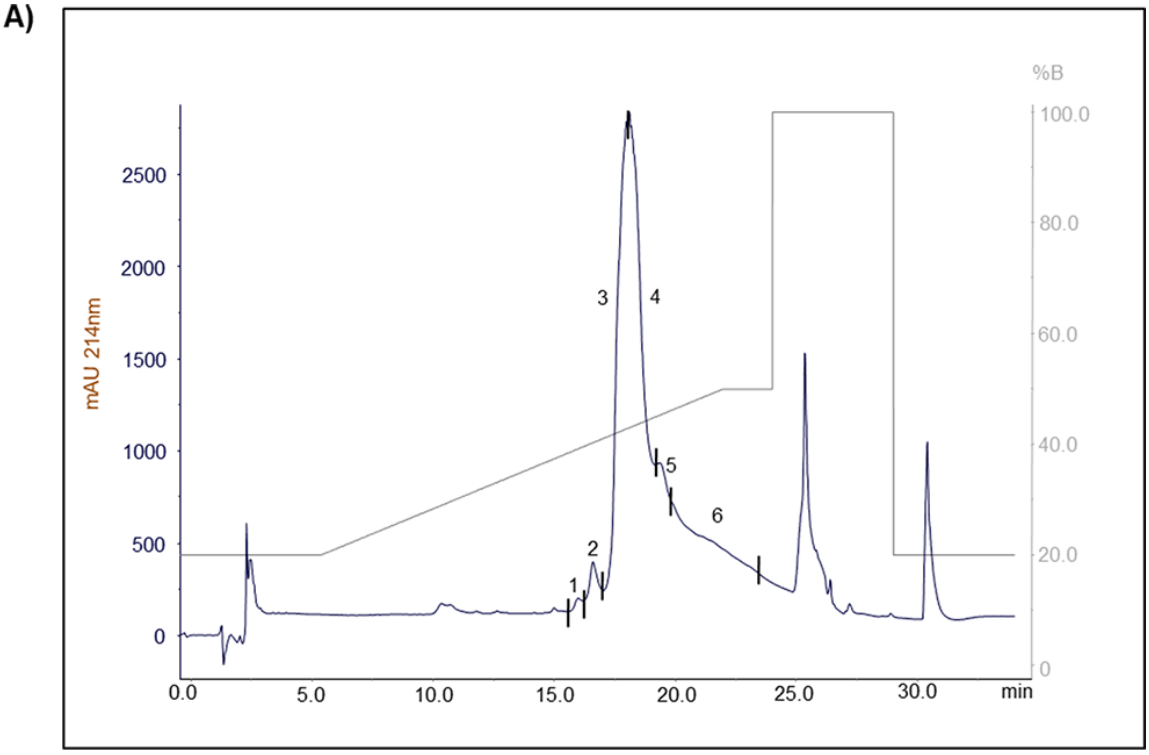

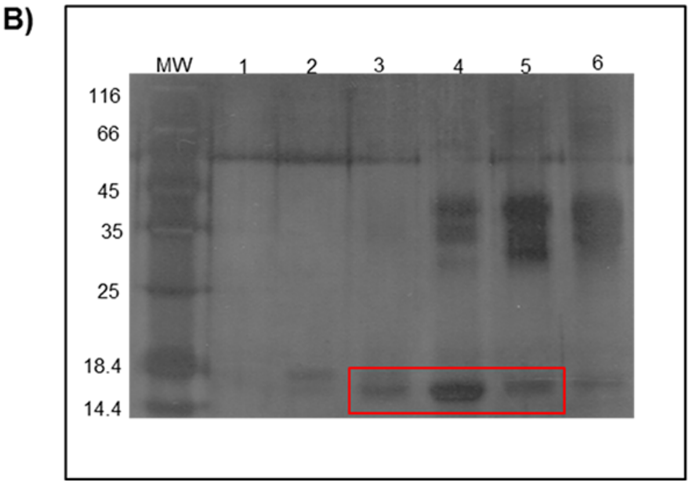

Figure S3. A) Third fractionation for purification of *Thalassophryne maculosa* TmC4-47.2 toxin. Fractions 2s, 2d and 3 were mixed and 1 mg.mL^{-1} of the pool was applied to a reversed-phase analytical C8 column coupled to a high-pressure liquid chromatography (HPLC) system. The gradient used was 20 to 80% buffer B over 35 minutes under 1 mL.min^{-1} flow rate. The absorbance was monitored at 214 nm. B) The protein content of fractions 1, 2s and 2d was evaluated by 12% SDS-PAGE electrophoresis on polyacrylamide gel (10 µg/well). MW corresponds to molecular mass markers and the bands highlighted in red refer to TmC4-47.2 toxin.
